# Factors Influencing Motivation and Engagement in Mobile Health Among Patients With Sickle Cell Disease in Low-Prevalence, High-Income Countries: Qualitative Exploration of Patient Requirements

**DOI:** 10.2196/14599

**Published:** 2020-03-24

**Authors:** David-Zacharie Issom, André Henriksen, Ashenafi Zebene Woldaregay, Jessica Rochat, Christian Lovis, Gunnar Hartvigsen

**Affiliations:** 1 Division of Medical Information Sciences Geneva University Hospitals Geneva Switzerland; 2 Faculty of Medicine University of Geneva Geneva Switzerland; 3 Department of Community Medicine UiT – The Arctic University of Norway Tromsø Norway; 4 Department of Computer Science UiT – The Arctic University of Norway, Norway Tromsø Norway

**Keywords:** mHealth, wearable devices, self-management, sickle cell disease, patient engagement, adoption, motivation, user computer interfaces, health behavior, persuasion

## Abstract

**Background:**

Sickle cell disease (SCD) is a hematological genetic disease affecting over 25 million people worldwide. The main clinical manifestations of SCD, hemolytic anemia and vaso-occlusion, lead to chronic pain and organ damages. With recent advances in childhood care, high-income countries have seen SCD drift from a disease of early childhood mortality to a neglected chronic disease of adulthood. In particular, coordinated, preventive, and comprehensive care for adults with SCD is largely underresourced. Consequently, patients are left to self-manage. Mobile health (mHealth) apps for chronic disease self-management are now flooding app stores. However, evidence remains unclear about their effectiveness, and the literature indicates low user engagement and poor adoption rates. Finally, few apps have been developed for people with SCD and none encompasses their numerous and complex self-care management needs.

**Objective:**

This study aimed to identify factors that may influence the long-term engagement and user adoption of mHealth among the particularly isolated community of adult patients with SCD living in low-prevalence, high-income countries.

**Methods:**

Semistructured interviews were conducted. Interviews were audiotaped, transcribed verbatim, and analyzed using thematic analysis. Analysis was informed by the Braun and Clarke framework and mapped to the COM-B model (capability, opportunity, motivation, and behavior). Results were classified into high-level functional requirements (FRs) and nonfunctional requirements (NFRs) to guide the development of future mHealth interventions.

**Results:**

Overall, 6 males and 4 females were interviewed (aged between 21 and 55 years). Thirty FRs and 31 NFRs were extracted from the analysis. Most participants (8/10) were concerned about increasing their physical capabilities being able to stop pain symptoms quickly. Regarding the psychological capability aspects, all interviewees desired to receive trustworthy feedback on their self-care management practices. About their physical opportunities, most (7/10) expressed a strong desire to receive alerts when they would reach their own physiological limitations (ie, during physical activity). Concerning social opportunity, most (9/10) reported wanting to learn about the self-care practices of other patients. Relating to motivational aspects, many interviewees (6/10) stressed their need to learn how to avoid the symptoms and live as normal a life as possible. Finally, NFRs included inconspicuousness and customizability of user experience, automatic data collection, data shareability, and data privacy.

**Conclusions:**

Our findings suggest that motivation and engagement with mHealth technologies among the studied population could be increased by providing features that clearly benefit them. Self-management support and self-care decision aid are patients’ major demands. As the complexity of SCD self-management requires a high cognitive load, pervasive health technologies such as wearable sensors, implantable devices, or inconspicuous conversational user interfaces should be explored to ease it. Some of the required technologies already exist but must be integrated, bundled, adapted, or improved to meet the specific needs of people with SCD.

## Introduction

### A Tsunami of Mobile Health Apps for Chronic Disease Self-Management

Mobile health (mHealth) apps are flooding the app stores, with 200 new apps each day [1]. Many apps can significantly improve health outcomes [2,3] by supporting people with diverse medical conditions [4]. However, little is known about the app usage frequency and long-term engagement among chronic patients [5,6]. Indeed, Robbins et al [7] underlined that people who would most benefit from such apps underuse them. To promote mHealth usage, app developers need to comprehend what could motivate patients to engage in mHealth [8]. The theory of reasoned action [9,10] demonstrates that the likelihood to engage in a specific behavior is a function of the motivation to perform it. Authors, such as Coa et al [11], confirmed that calculating baseline motivation levels could predict retention rates for apps. To influence people’s motivation through persuasion rather than coercion, the field of persuasive technology offers novel user-centered approaches (ie, cocreation) [12-18].

### The Case of Sickle Cell Disease: One of the World’s Most Neglected Chronic Diseases

In this paper, we focus on sickle cell disease (SCD). SCD is the most common monogenic blood disorder in the world. Studies approximate 400,000 neonates per year [19,20] and between 25 and 100 million patients worldwide living with the disease [21,22]. In this study, we specifically target populations from low-prevalence areas of high-income countries. In these settings, the disease is underresourced, research-derived evidence is lacking, and patients are particularly isolated [23].

SCD is still a serious illness. Hemolytic anemia and vaso-occlusive pain crises (VOCs) are the hallmarks of the disease [24]. Patients may suffer severe and potentially lethal complications [25]. Hydroxyurea, the preferred disease-modifying treatment, is underutilized and not effective for every patient [26]. Furthermore, the only curative option, bone marrow transplant, is largely inaccessible [27].

However, public health interventions such as regional screening programs, preventive care, coordinated care and comprehensive care plans have been introduced in the major regions of high-prevalence, high-income countries [28]. These interventions drastically reduced early childhood mortality and made SCD shift to a chronic disease of adulthood [29].

Nevertheless, such programs have not been implemented widely and remain virtually absent in most low-prevalence, high-income countries [20,30]. In addition, there are few trained physicians and access to specialized health care is suboptimal [31-33]. Indeed, patients with SCD are particularly prone to confront stigmatization, to suffer unequal treatment, and to experience health care injustice (ie, perception as drug seekers) [33-35]. This often leads to mistrust between patients and health care providers [36,37]. As several studies demonstrated [38-44], when patients arrive in emergency departments, the lack of objective hematological findings and little awareness of those findings make health care providers suspicious of the veracity of a VOC. This distrust makes the SCD community infamously difficult to recruit in research initiatives and hard to engage in interactions with medical providers, and the distrust also lowers adherence to medical recommendations [36,45-48].

Consequently, most patients are left to self-manage and rely on poor-quality health care, and they report low levels of quality of life [49,50].

### Persuasive Mobile Health Interventions to Support an Undersupported Population

To make matters worse, as several studies demonstrated [51,52], self-care management is challenging for people with SCD. Indeed, managing the numerous potential precipitating factors of VOCs requires high levels of self-efficacy [52-55]. Well-known triggers [56] include inadequate diet, stress (ie, exertional, oxidative, and psychological), infections, inflammations, acidosis, dehydration, fatigue, chronic hemolysis, hypoxia, smoke inhalation, alcohol intoxication, pregnancy, and environmental factors (ie, altitude, pollution, extremes of temperature, climate, and wind speed). As a consequence, SCD self-care management practices require patients to pay special attention to everything. In a usual day, extreme temperature changes, bad weather, and high altitudes should be avoided; eating behaviors should be optimized with regular hydration; exercise should be moderate and not exhausting; rest should be sufficient; prescribed drugs should be taken; and stress should be managed and avoided as much as possible [50,57-59]. Finally, as in all diseases, socioeconomic factors such as education levels, occupation, or income play an important role in empowering patients [60,61].

mHealth apps, because of their relatively low cost and wide reach, could offer a potential route to support patients’ numerous self-management tasks [62]. Little work has been done to design tailored mHealth interventions for the comprehensive self-management needs of patients with SCD [63,64]. Today, most existing apps and research focuses on medication adherence [65]. Consequently, solutions encompassing the multiple components of SCD self-management are absent. Shah et al [66] suggested that patients with SCD could be interested in such tools. However, as with other chronic diseases, little is known about those patients’ mHealth app adoption and long-term engagement.

This paper is the last component of a study from which preliminary results have already been published [67]. This prior publication was the first to elaborate on mHealth long-term engagement among patients with SCD. The authors explored common motivational patterns for mHealth use between patients with SCD, patients with diabetes, and *healthy* people.

This paper focuses on adults with SCD living in low-prevalence areas of high-income countries. It was aimed to assess patients’ requirements in terms of value-adding digital health tools and to guide the development of future mHealth interventions that people with SCD would want to use.

## Methods

### Inclusion Criteria

To be part of the study, applicants had to be diagnosed with SCD or be the caregiver of a person with SCD. Participants had to be at least 18 years old and able to understand French or English. People who had been cured (ie, bone marrow transplantation) were excluded.

### Recruitment

The sample for this study was a convenience sample from Switzerland and Norway, two very low-prevalence, high-income countries. Indeed, compared with an average in the European Union of 2.5 cases in 10,000 people [68], these two countries have less than approximately 1 to 4 cases per 100,000 people [69-71] and a total of approximately 100 adult patients with SCD. We recruited participants through the national patient associations’ online support groups. In all, 64 individuals with SCD were invited to participate. One week after the initial invitation, nonresponders were sent a reminder.

### Instrument

The first author (DI), an expert patient, conducted most of the semistructured interviews using the guide presented in Table 1. The choice of an *insider* interviewer was made to build a trusted, warm, and open rapport with the interviewees and to maximize the reception of honest and open responses. In addition, the interviewer was already familiar with some participants. JR conducted the test interviews. Both interviewers have many years of interview experiences. Interviews were conducted in locations convenient and comfortable for the participants (ie, university and private address).

The interview guide was developed previously as a joint effort by all coauthors [67]. It was divided into five themes: (1) preliminary questions, (2) goals, expectations, and attitudes, 3) electronic health literacy and data integration, (4) wearables and sensors, and (5) data sharing. Data saturation was reached and determined by no new information emerging after conducting interviews with all participants. Interviews were audiotaped and lasted approximately 60 min. Questions were open ended, and discussions were conducted flexibly. Questions were ignored or adapted relative to the context.

**Table 1:** Overview of the questions asked during the interviews.

Themes	Questions
Preliminary questions	What is most important for you in your life? If you had access to a new health technology, which purpose or features should it have?
Goals, expectations, and attitudes	What motivates and demotivates you to access Web-based health information? What are the most difficult things about your self-care? What could help you become more autonomous with your self-care?
Electronic health literacy and data integration	Have you ever used an app that collects health data? What factors would discourage you from using such an app?
Wearables and sensors	What indication would you expect from wearable sensors for health self-monitoring? What is the most valuable indication you would want from devices collecting your data?
Data sharing	What would you share with other patients, caregivers, and doctors and why? What feedback should be provided by the system?

### Data Analysis

First, transcriptions of the resulting interviews were deidentified. Second, an inductive thematic analysis was conducted using the guidelines and checklist from Braun et al [72]. Codes were extracted by reading the interviews recursively. Third, emerging patterns were clustered together and checked for variability and consistency. Themes were interpreted by reading the codes back and forth. Once saturation was reached, themes were mapped across the capability, opportunity, motivation, and behavior model (COM-B), hub of the behavior change wheel (BCW) framework [73]. The BCW is a fairly recent, theory-driven approach that helps to design health interventions for preventive care [73]. With the specific reading grid it provides, it allows us to identify barriers and enablers of engagement in any intervention and in our particular case, to identify factors that, if implemented together, may elicit the long-term engagement and user adoption of mHealth apps [74-76]. Transcripts were organized and coded using ATLAS.ti version 8.3.20.0 (ATLAS.ti Scientific Software Development GmBH, Berlin, Germany).

Finally, to make it easier for software developers to comprehend, themes were categorized into functional requirements (FRs) and nonfunctional requirements (NFRs) [77]. In software engineering, FRs are descriptions of the specific behaviors and functions of an information system. They make explicit the features a software should offer to the end user. In other words, it describes what the system should do [78]. NFRs are descriptions of how the system should operate, which is not linked to the functionalities. To put simply, NFRs define how the system should be (eg, responsive, intuitive, fast, and secure).

### Ethics and Study Approvals

The Norwegian Regional Committees for Medical and Health Research Ethics and the Swiss Regional Research Ethics Committee approved the study protocol and interview questions. As required by committees, all participants gave informed consent before the interview, and responses were anonymized.

## Results

### Participant Characteristics

A total of 11 participants (7 males and 4 females) agreed to join the study. In all, 8 patients and 3 caregivers took part in the study, but 1 patient withdrew because of a VOC. This led to a total of 7 patients and 3 caregivers participating in the study. Patients were aged between 21 and 55 years. A total of 7 participants were residing in Switzerland, 3 in Norway, and 1 was partly residing in the Democratic Republic of the Congo and in Switzerland. Table 2 presents the demographics of the population studied.

**Table 2:** Distribution of characteristics of the studied sample (N=10).

Characteristics of respondents		Value
Gender, male, n (%)		5 (50)
**Age (years)**		
	Mean (SD)	35.6 (9.41)
	Median	37
**Country of residence, n (%)**		
	Switzerland^a^	7 (70)^a^
	Norway	3 (30)
	Democratic Republic of the Congo^a^	1 (10)^a^

^a^One respondent was partly residing in the Democratic Republic of the Congo and in Switzerland.

### Interview Results

This section shows extracted themes from the interview data. The themes were classified into 31 FRs and 30 NFRs. We organized them with the COM-B framework and illustrated them with quotations from interviewees. Table 3 presents some of the most frequent themes that appeared during interviews. Each theme is an FR or an NFR and belongs to a COM-B system category. In the table, themes are sorted by COM-B system attribute, type of requirement, and then by the number of quotes. The complete list is available in Multimedia Appendix 1.

**Table 3:** Overview of potential influencers of adoption through the capability, opportunity, motivation, and behavior (COM-B) lens.

Requirement	Quotes	COM-B system	Type
Prevent crises by avoiding symptoms	12	Automatic motivation	Functional
Family and social community support (shareability)	15	Automatic motivation	Nonfunctional
Gain more control on disease through daily self-care support	28	Physical capability	Functional
Limit management	9	Physical capability	Functional
Importance of information trustworthiness	17	Physical opportunity	Nonfunctional
Invisibility or inconspicuousness	9	Physical opportunity	Nonfunctional
Similarity with messaging apps	8	Physical opportunity	Nonfunctional
Automatic reminders and automatability	5	Physical opportunity	Nonfunctional
Simplicity	3	Physical opportunity	Nonfunctional
Learn trigger factors	14	Psychological capability	Functional
Predict health outcomes	11	Psychological capability	Functional
Receive threshold alerts	10	Psychological capability	Functional
Feedback on self-care practices	17	Psychological capability	Nonfunctional
Customizable	8	Reflective motivation	Nonfunctional
Privacy	4	Reflective motivation	Nonfunctional
Learn what other patients do	14	Social opportunity	Functional

#### Physical Capability

All participants were concerned about not being able to better predict the onset of VOCs or avoid chronic complications. Another point to consider is that many differentiated general daily self-management skills (ie, pain management) from preventive care (ie, symptoms prevention). As one patient said:

It’s difficult when the situation changes in a short time and prevents you from being active, without warning. When suddenly you cannot do anything.

Patient 5, 49 years

In addition, a couple of interviewees said they would want novel technologies that automatically regulate their hematologic parameters (ie, hemoglobin concentration, leucocyte adherence to vascular endothelium) [79]. Accordingly, a participant proposed a very innovative solution:

I would like nanorobots that help my blood stay normal, self-regulate.

Patient 9, 30 years

#### Psychological Capability

The majority of participants desired to receive feedback about SCD self-care tasks. Most emphasized the difficulty of managing their own limits. Some wished to be warned before exceeding their physical limitations. Namely, an interviewee proposed:

A sensor that informs me if I can go into the water or…when I play sport, a sensor that allows me to know what I have to limit, if there is a threshold not to exceed.

Patient 1, 21 years

Importantly, most of participants suggested that such warnings should be detected with wearable sensors, releasing their cognitive load. This can be illustrated by the following quote:

Something that shows my limitations. For example, I would not want something that tells me 'if you go on like this, you're going to have a crisis'. But something configurable. Because, I like pushing my limits. For example, when I’m walking, well... first it must realize that I’m walking, but it could ask me how I feel, learn my limits.

Patient 9, 30 years

#### Social Opportunity

For most participants, learning about other patients’ self-care practices was a very important concern. A majority of the participants said they would like to share their own experiences on digital platforms. For example, one participant said:

An indication of good health. And see what influences what in whom. Compare what people do to feel good.

Caregiver 1, 37 years

Many participants stressed the importance of social support. Some stated that mHealth could help them communicate their needs. As one participant suggested:

Help family members to better understand the problem, but also for crisis prevention, teach people how to manage the disease.

Caregiver 3, 49 years

A majority of participants reported using social media for information sharing, communication, or entertainment. Only a minority of them used social media to get health information. Many participants deplored the absence of mechanisms to easily access and control the quality of information. As one participant said:

How to check the veracity of information sources and information. How to demonstrate that it is validated information. We can already see that fake news may surpass the traditional media. So it's a sensitive topic.

Patient 2, 37 years

#### Physical Opportunity

For several interviewees, it was crucial to receive trustworthy information. Many suggested that wearable sensors could support this. As one participant highlighted:

Indications that support my decisions, based on sensor data or collected data. An alert, a barometer that allows me to refocus and would say: “be careful, be careful, your temperature is high, your hemoglobin is low, your CRP is high, your oxygen is low, you should rather be at this level.”

Patient 9, 30 years

Several participants stated that information overload would demotivate them for long-term engagement, specifying that mHealth apps should be as discreet as possible. Furthermore, most participants recognized the potential usefulness of notifications, but only if discreet and not disturbing. As one participant stressed:

Yes, but not frequently, not something that screaming “you're sick! you're sick!”

Patient 7, 55 years

Notwithstanding, the majority of participants preferred user interfaces that are simple to use and do not require a high learning curve. Some participants believed messaging software was the best inspiration because of its intuitive interface. As one caregiver said:

A technology that is easy to use, user-friendly for the digital illiterate. A system using widespread technologies. A system, an application that has a long life cycle. Proven technology like messaging, a simple thing that everyone can understand.

Caregiver 2, 37 years

#### Motivation

##### Reflective Motivation

Importantly, a majority of the participants were not using mHealth apps. One of the 10 participants reported using Apple Health for menstruation management, hydration, and physical activity management. Two participants reported using Samsung Health for blood oxygen recording and physical activity management. Another point to consider is that all participants expressed a lack of specificity and adaptability to SCD particularities in the usual health apps (ie, normal values for people with SCD are the values indicating a disease in healthy people). As one participant stressed:

Things should be adapted, for instance since oxygen or arrythmia are usual for SCD. It should be adapted. It's difficult to compare to normal people.

Patient 6, 35 years

Privacy issues were also a fundamental concern for most interviewees. Many feared to lose control on their data. As one participant said:

I am not against connected devices, Cloud… I just do not want to be found and get problems.

Patient 2, 37 years

Finally, most participants preferred customizable information systems. As one interviewee said:

I want to be the decision maker of what I share. I want to control what I receive.

Patient 9, 28 years

##### Automatic Motivation

The three main motivators identified were (1) *strengthen social support*, (2) *prevent VOC*, and (3) *reduce the limitations to functioning and independent living*. Regarding the first motivator, participants wished to be able to enjoy with their families and give back to their communities. As one participant said:

My daughter is the most important thing in my life, because of that, I must not forget my health. If I’m not happy she’s not happy.

Patient 7, 55 years.

Regarding the second motivator, all participants stated their desire to prevent the excruciating pain crises. As one participant said:

I want to be able to regulate, to avoid health problems before they appear, to prevent rather than to cure.

Patient 4, 29 years

More importantly, most participants stressed the importance of living as normal a life as possible and stay in good health. This can be illustrated by the following quote:

I am particularly motivated by the desire to live well, to be like everyone else. For my self-esteem, to show that I am able to do the same things as a normal person, and even better.

Patient 9, 28 years

## Discussion

### Principal Findings

To the best of our knowledge, this is the first study to explore factors that could influence the long-term engagement with mHealth interventions of adults with SCD living in low-prevalence, high-income countries. As a result, interviewees described how mHealth could benefit their life and detailed what could increase their long-term engagement and motivation toward the mHealth app usage.

### Functional Requirements in the Prism of the Capability, Opportunity, Motivation, and Behavior Framework

To maximize the chances of eliciting behavior change and engagement, as many patient requirements as possible should be implemented. In other words, providing patients with information or alerts is not sufficient to lead to behavior change. However, through simultaneously implementing other features such as therapeutic education (eg lessons and accompaniment), specifically designed wearable devices could maximize engagement. In the following sections, we propose potential solutions and summarize what needs to be done before being able to develop specifically designed wearable devices.

#### Increasing Physical Capability: Regulate Hematological Parameters

Patients suggested how pioneering technologies such as blood-regulating nanorobots could inspire future pharmaceutical research or stimulate patient-led research initiatives [80]. By altering hematological parameters, some innovative pharmaceutical compounds are already aligned with patients’ suggestions [56,81]. For instance, recent clinical trials of crizanlizumab, an antibody acting against endothelial adhesion, have shown a significant reduction in the frequency of VOCs [82]. However, this substance requires frequent intravenous injections. Therefore, knowing the current underutilization of orally administrated therapies such as hydroxyurea, intravenous therapies bring a supplementary barrier.

In the meantime, although nonpharmaceutical strategies based on information technologies could inform patients on how to adapt their behaviors (ie, dietary change) to alter blood parameters (ie hemoglobin levels, oxygen levels, and inflammation) [83-86], novel devices, inspired from other clinical populations, could be created. This can be illustrated by what is happening in the Diabetes Do It Yourself (DIY) community [87] (ie, #WeAreNotWaiting and #DIYPS). Here, impatient patients self-organized to hack blood glucose–monitoring system and insulin pump. They proceeded to create a system that can, after clever calculation, automatically inject the needed dose of insulin. In comparison, one could foresee similar initiatives of DIY solutions supporting SCD self-care practices. In particular, as a reminder, anemia and oxygen desaturation are common complications among people with SCD. As oxygen delivery by hemoglobin increases when the number of red blood cells and the hemoglobin level increases, one could imagine creating a closed-loop system using a wearable hemoglobin meter or a blood oxygen meter. This could subsequently be combined with an autoinjector filled with ultrashort-term anemia-reducing treatments [88] (eg, erythropoietin alpha, vitamin B_12_, oral folic acid, and voxelotor [89]).

Nonetheless, it is important to consider that a significant amount of work is required before such a system could be created and made accurate or safe. Even if bypassing any approval from a health authority (eg, Federal Drugs Administration), implantable autoinjectors and specific biomonitoring devices would have to be engineered. Then appropriate software would have to be created to be able to analyze significant quantity of patient-generated data. Indeed, to be accurate and effective, such algorithm should be trained on a high quantity of data. Afterward, the data-driven algorithm should be able to actuate the injection of individualized doses of the adequate substances. All of this requires a tremendous amount of skilled bioengineering work. Another point to consider is the lower socioeconomic status of members of the SCD community [90] and the low awareness of SCD among the general population, especially in high-income, low-prevalence countries. This mechanically leads to a limited number of patient innovators and interested independent researchers. In consequence, few people would be skilled to build such a specialized system.

Therefore, the SCD community could start with less invasive, less complex, but also less cognitively unloading solutions. A simpler system could be proposed to prompt patients to ingest relevant drugs or dietary supplements (eg, anti-inflammatory drugs and antioxidants), after a biosensor [91] (eg, blood oxygen and inflammation) detects a threshold. In addition, an mHealth app could send an alert. On the one hand, medication adherence would then become an issue to overcome, on the other, if the medication is taken, the effect of the substance would be delayed compared with a direct subcutaneous injection.

To summarize, the most effective solution using today’s technology would be a closed-loop system with an autoinjector and smart algorithm, functioning without any patient intervention, but there is still a long and challenging way to go before such a system could be built.

#### Increasing Physical Capability: Stop Pain Fast

Quick pain relief was a very important concern for most interviewees. In the current VOC self-management, pain crises are treated at home with oral painkillers [92,93]. When oral drugs are no longer sufficient or when complications surge, patients need to visit emergency departments to receive acute care [38]. However, despite being aware of the several challenges they would face in emergency departments (ie, long waiting times, recurrent stigma, unrelieved pain, and prolonged hospitalizations), many patients choose to postpone the admission until pain becomes totally unbearable [94,95].

These challenges could partly explain why interviewees were highly motivated by mHealth interventions that could help them relieve their pain as fast as possible. To this end, novel sensors and software measuring pain levels through physiological signs or electrical signals [96] could be combined with implantable pumps for intrathecal opioid therapy or subcutaneous injections [97]. However, to decrease cognitive load and avoid patients to calculate themselves how much they should inject, smart, accurate, safe, and individualized algorithms would have to be developed.

In the United States, one mHealth app helps children and adolescents with SCD to inform family, physicians, or friends about their health status [98]. Recently, innovative digital health interventions have been deployed to facilitate emergency care process [99]. We could also imagine apps that allow sufferers to support and motivate each other or to come together to share their experience of treatment efficacies [100,101]. Existing mHealth apps for pain management could be adapted to SCD [102]. Finally, virtual reality could be used as a new option for pain relief through patient distraction [103].

#### Increasing Psychological Capability: Quality Feedback on Self-Care Practices

The complexity of SCD self-care tasks demands various skills (eg, high cognitive capabilities and good disease-specific knowledge) [59]. The literature has shown that only 1% of SCD patients were able to master them [104]. Therefore, it is easy to understand why most interviewees desired to receive feedback on their self-care practices.

For instance, a release of the cognitive load could be partially decreased by using a natural user interface (eg, text, voice, mixed reality, and augmented reality), gamification items [105], and simple data visualization [106-109] when providing patients with targeted information. The technology exists but needs to be integrated, adapted, bundled, and improved [110-112]. Inspiration could be taken from already existing systems for people with diabetes [113,114].

Furthermore, studies have shown that behavior change techniques for self-management such as health coaching could improve the outcomes important for patients, such as self-efficacy, self-management, or medication adherence, by 80% [115-118]. For instance, collecting physiological and behavioral data, such as eating behaviors and oxygen levels, and then automatically reporting them to an SCD expert could allow the latter to provide advice directly in the mHealth app. This would not require overcoming many barriers as data could already be collected through fitness and wellness apps and smartwatches. However, the biggest challenge lies in the development of smart algorithms and methods to allow the automated interpretation of such individualized and heterogenous data [119]. In the meantime, coaching and high-quality electronic patient decision aids could be created to structure information and help patients make informed decisions [120-122].

Regarding disease-specific knowledge, studies have reported that many patients did not follow medical recommendations [45] but may still search for health information on the Web. When the quality of information is poor, disparities in health information accessibility are created [123,124]. Frost et al [125] have already discussed the promises of Web-based health information for people with SCD, and Breakey et al [126] argued that information was not always adequate and of good quality.

Consequently, the use of artificial intelligence [127] with evaluation criteria, such as Health On the Net code [128] or the DISCERN [129] framework, could facilitate the provision of quality material to patients. Finally, content constructed with the help of patients with SCD and using consumer health vocabulary, simple patient language, and thesauri could improve the communication of health information and adequacy [130,131].

#### Increasing Physical Opportunity: Receive Alerts When Reaching Own Physical Limits

Already struggling with normal life challenges, several interviewees strongly desired help to manage the potential triggers of VOC. However, objective laboratory, clinical, hematological, biochemical, and rheological data are not easy to self-monitor [41-43,132-134]. Existing noninvasive sport-related wearable device technologies could help monitor some markers of hematological parameters [96]. For instance, sport watches with pulse oximeters [135], connected bottles, oxidative stress monitor, and pH meters [136,137] could be used. To this end, smart algorithms could be developed to provide patients with individualized feedback.

Studies have shown that poor physical functioning was frequent among people with SCD, making their participation in sports difficult [138,139]. mHealth interventions specifically tailored for physical activity support could be developed to assist people with SCD. Accurate sensors could be integrated during physical activity (ie, exhaustion) or after exercise (ie, recovery) [140,141].

#### Increasing Social Opportunity: Learn What Other Patients Do

One of the most reported motivational factors was the desire to learn from other patients. This consideration is coherent with the stigma and isolation faced by patients with SCD living in low endemic areas [142]. This concern is also consistent with the general lack of educational interventions [143] and the limited availability of specialized health care providers [144]. It is well known that patients with a chronic disease hold nonnegligible experiential knowledge (eg, effective dietary supplements and where to find them, tips) and often share it on social networks [145-147]. However, the knowledge available on these platforms is difficult to extract, the quality is difficult to guarantee, and the information is often unstructured, hence difficult to mine [148].

Well-funded and organized social networks such as Patients Like Me or Diabetes online support groups (eg, TuDiabetes, glu, and Diabetes Daily) could serve as inspiration to structure patients’ experiential knowledge [149]. However, in comparison to the total SCD population, few patients with SCD use online support groups. A study by Ragnedda et al [150] has demonstrated that socially disadvantaged groups (eg, gender, ethnicity, and disability) tended to use the internet less than more advantaged groups. Conversely, a study by Issom et al [151] suggests that patients with SCD would be willing to use such online support groups if the quality of information is ensured and if it is specifically tailored for people with SCD.

Consequently, SCD-specific online support groups such as OneSCDVoice [152] could be turned into persuasive social networks with questions and answers, ensuring the medical accuracy of patients’ shared experiences [153]. In addition, artificial intelligence techniques could be used to help detect low-quality information [154]. Another issue is the multitude of SCD online support groups (eg, hundreds of Facebook groups and various websites). This spreads the information, fragments messages, and complicates access to experiential knowledge. In addition, as people with SCD and their caregivers have low educational levels [61], their organizational skills and digital literacy are reduced, subsequently hindering their access to online health information.

#### Main Motivations: Live a Normal Life and Learn to Avoid Symptoms

A majority of participants stated that their highest motivation would be to be asymptomatic. When bone marrow transplant is not possible, or when hydroxyurea does not significantly reduce symptom frequency, alternative treatment options such as self-management interventions, chronic transfusions, or red blood cells exchange could be proposed [155]. Transfusions have shown to significantly reduce the frequency of VOCs but require high quantities of rare phenotype blood. In addition, phenotype matching is difficult in high-income, low-prevalence areas. Self-management interventions require a high level of self-efficacy. Electronic patient decisions aids (ePtDAs) could be helpful. Indeed, ePtDAs have proven effective in engaging patients in self-care processes and helping patients choose alternative treatment options [156]. To date, there are no such tools for SCD. However, a study by Kulandaivelu et al [157] showed that people with SCD asked for such help.

To push for the development of such systems, patients would require to be more aware of novel treatment options, innovative solutions, or self-management support possibilities. As a result, they could better organize to create awareness among potential payers (eg, philanthropists and pharmaceutical companies) who could help finance the development of such advanced solutions. However, this remains a challenge for the socioeconomically disadvantaged majority of people with SCD [61].

### Nonfunctional Requirements

This section discusses the most frequently reported NFRs by the interviewees. NFRs important for the patients are crucial when developing software [158]. A mismatch between them and the final product could lead to low adoption rates and discourage app usage.

#### Automatability

Participants clearly preferred automatic health data acquisition rather than manual data entry. To date, there is no SCD-specific mHealth intervention using automated data capture. However, data from electronic health records, smartphone sensors, or wearable devices [159] could be automatically collected in future mHealth interventions for people with SCD [160].

#### Invisibility

Invisibility or inconspicuousness is the ability of a system not to attract attention. As many interviewees reported and consistent with existing literature [161], mHealth interventions are more likely to be adopted if they clearly reduce the inconvenience and burden of self-management tasks, while being discreet. Indeed, bulky and inelegant wearable devices could indicate to other people that wearer has a disease. Implantable devices, discreet patches, and wearables with subtle design or integrable into daily life objects (eg, contact lenses, implants, and bottles) could be preferred [162-165].

#### Similarity With Messaging Apps

More than half of the respondents called for an mHealth intervention that provides a user experience similar to that provided by the apps they are using the most (ie, messaging apps). Only few studies have explored conversational designs for mHealth interventions. However, some conversational user interfaces have been successfully used to reduce obesity or as support for mental health interventions [166,167]. Such systems have been already shown to encourage behavior changes and have high levels of acceptability. This could encourage similar designs in future studies for patients with SCD.

#### Shareability

In the context of Web-based health information, shareability [168] is the capacity of patient-generated health data to be shared with third parties. By allowing patients to share their knowledge, mHealth apps could highlight valuable information that clinicians cannot offer [169] and ease social support [170]. As for people with rare diseases, question and answer social networks [171] could be leveraged [172].

#### Privacy

In the mHealth context, privacy is the ability of a patient to protect information about their medical condition [173]. Being aware of the rarity of SCD in their countries, interviewees were particularly attentive to this issue. Participants emphasized that control should be given on what they share and to whom. Studies for other chronic diseases showed that privacy is a very important aspect [174]. Answering this concern by taking into account regulations (ie, General Data Protection Regulation and Health Insurance Portability and Accountability Act) when designing mHealth interventions would be key to reduce poor adoption [175]. Here, existing compliant data management platforms with dynamic consent management [176] or personal data cooperatives [177] could be used. Furthermore, novel deidentification approaches could help implement this NFR important for the patients [178].

#### Customizability

To maximize acceptability and inconspicuousness, most participants proposed the customization of the timing of delivery of push notifications. This finding is aligned with a study by Morrison et al [179] suggesting that notifications with tailored timing could enhance exposure to mHealth interventions. However, several patients stressed their dislike of recurrent notifications (ie, water intake), saying it would remind them of their disease. This is aligned with a study by Bidargaddi et al [180] suggesting that notifications should be sent at midday or on weekends. The truth probably lies in between, where a system could allow patients to choose from a range of predefined settings.

Finally, as the interviews demonstrated, patients did not think that existing health apps where adapted to their needs. As a result, any new digital health solution for people with SCD should include patients in every phase of the development and focus on SCD particularities. Finally, the developed system should be strongly marketed as a patient-centered solution.

### Lessons Learned

Our findings highlighted the participants’ very clear expectations toward mHealth apps. Respondents seemed undeniably motivated to use *an invisible technology* that would accompany their self-care practices (ie, personalized feedback). Participants were very critical toward privacy issues and information quality. Given the numerous and complex day-to-day self-care management tasks that patients with SCD face and taking into account the isolation of living in low-prevalence, high-income countries, it appears that, in order to get long-term engagement and adoption, mHealth apps must add clear value and be particularly tailored to patients’ needs.

#### From Requirements to Successful Implementation

For such an mHealth solution to have an impact once implemented, quality and perceived value must be distinguished. However, the digital health world still lacks a standardized mechanism for health app quality evaluation and certification [181]. There is no consensus for guidelines, nor clear criteria to help recognize what a quality mHealth app is. For instance, people with diabetes, with more than 300 apps on the Google Play Store, can easily struggle to find what apps they should download. Some app curation websites [182] help end users to find health apps ranked by quality. However, the analytic methods vary for each website. For instance, in the United Kingdom, the National Health Service Digital and National Institute for Health and Care Excellence has chosen clinical effectiveness, regulatory approval, clinical safety, privacy and confidentiality, security, usability and accessibility, interoperability, technical stability, and change management as criteria. Other websites could use other criteria, for instance apps for which there is published medical evidence.

As a result, app developers should make an effort to meet requirements important for the patients and thrive to meet as many quality indicators as possible. This can be summarized with the following steps:

- Take a patient-centered approach: make sure the app improves patient outcomes; and the app is validated in terms of clinical outcomes.
- Offer a real solution to self-management problems faced by patients: the app should meet patients’ requirements and do better than any alternative and should be cheaper.
- Be compatible with the existing health care information system infrastructure: implement interoperability standards by design; and answer privacy and safety requirements.

### Key Points

Nevertheless, it is clear that there is no *invisible technology* yet to support the self-care practices of patients with SCD. However, the various technological pieces needed to build such a ubiquitous system are largely available today. Nonetheless, they are scattered, not bundled nor adapted to the specificity of SCD. The following steps summarize what needs to be done before implementing the key requirements:

- Disease-modifying functionalities (ie, pain relief and regulation of hematological variables) to reduce poor adoption rates by providing patients with continuous blood oxygen meters, specific autoinjectors, and smart software that includes algorithms that are able to manage and make sense of the big datasets generated.
- Targeted information (eg, alerts when approaching limits and access to other patients’ self-care practices) could influence the long-term engagement by providing patients with knowledge adapted to their health literacy levels, user interface matching their digital literacy levels, and information adapted to their disease-specific knowledge, and if necessary, offer assistance to interpret the information.
- Controlling data flows (eg, shareability, privacy, and quality information) could influence patients’ motivation to start using apps by storing data on personal data cooperatives, implementing existing interoperable standards, using algorithms, and hiring content moderators (eg, physicians and expert patients) to monitor content creation and the quality of information.

### Future Plans

Given the complexity of SCD self-management, supporting self-care needs important for the patients using mHealth interventions will be challenging. However, such systems will be key to fill the gaps in health care delivery service. Further work is needed to implement patient requirements. Prioritization could be done using participatory approaches. Tools such as the APEASE (Affordability, Practicability, Effectiveness/cost-effectiveness, Acceptability, Side-effects/safety, Equity) criteria, a set of benchmarks from the BCW framework, can be helpful to decide what content should be included and what intervention delivery strategies should be used. In addition to proven added value with the effective implementation of requirements important for the patients, other NFRs include affordability, practicability, effectiveness and cost-effectiveness, acceptability, side effects, safety, and equity.

In addition, Delphi surveys [183], a technique consisting of seeking experts’ views to obtain a level of agreement by transforming opinion into group consensus, could be sent to expert patients [121,184].

Finally, motivational factors will have to be assessed in the long run to maintain high user engagement levels. This could be done using tailored frameworks for mHealth engagement analysis such as the Analyzing and Measuring Usage and Engagement Data framework [185].

### Strengths and Limitations

The study has a number of strengths and limitations. Using the COM-B model to identify motivational factors is a relatively recent approach in the field of mHealth. However, in this example, the lens offered by the model was helpful to gain a full picture of patients’ motivations. In addition, this model allowed us to classify patient requirements into explicit categories and helped us to discuss potentially useful technologies to meet patients’ unmet needs.

The age range of the sample was large; consequently, younger patients may feel more comfortable using digital health interventions than older ones. In addition, our sample size approximates 10% of the adults with SCD living in the selected low-prevalence, high-income countries. Also, because of the notorious difficulty in enrolling patients with SCD in studies, we were surprised by this relatively high response rate. The fact that the main interviewer was an expert patient may have eased enrollment and facilitated trust building but also added a bias.

Finally, the study results could be affected by the recruitment criteria. As participants were selected inside an active SCD community, it is possible that those who volunteered to be interviewed were more in search of new coping solutions and had more positive views about the disease than those who declined or did not replied.

### Conclusions

As interviewees were particularly explicit in what could benefit them, this study provides initial insights on how to build mHealth apps that could engage particularly isolated SCD populations. The use of qualitative methods enabled in-depth exploration of interviewees’ responses. Furthermore, the BCW and, its hub, the COM-B model could be used as a robust framework to inform the development of future persuasive technologies for people with SCD. As patients highlighted, future research should focus on supporting their self-care decisions. Exploring the integration, adaption, or improvement of highly adopted mHealth interventions for other chronic diseases could be helpful.

## Appendix

Multimedia Appendix 1 Themes extracted from interviews.

## Abbreviations

BCW: behavior change wheel
COM-B: capability, opportunity, motivation, and behavior
DIY: Do It Yourself
ePtDA: electronic patient decision aid
FR: functional requirement
mHealth: mobile health
NFR: nonfunctional requirement
SCD: sickle cell disease
VOC: vaso-occlusive pain crisis
